# Readmission and emergency department presentation after hospitalisation for epilepsy in people with intellectual disability: A data linkage study

**DOI:** 10.1371/journal.pone.0272439

**Published:** 2022-08-01

**Authors:** Peiwen Liao, Claire M. Vajdic, Simone Reppermund, Rachael C. Cvejic, Tim R. Watkins, Preeyaporn Srasuebkul, Julian Trollor

**Affiliations:** 1 Faculty of Medicine and Health, Department of Developmental Disability Neuropsychiatry, Discipline of Psychiatry and Mental Health, University of New South Wales, Sydney, Australia; 2 Faculty of Medicine and Health, Centre for Big Data Research in Health, University of New South Wales, Sydney, Australia; 3 Faculty of Medicine and Health, Centre for Healthy Brain Ageing, Discipline of Psychiatry and Mental Health, University of New South Wales, Sydney, Australia; Radboud University: Radboud Universiteit, NETHERLANDS

## Abstract

**Background:**

Despite the high prevalence of epilepsy and multiple barriers to care in people with intellectual disability, the risk of returning to hospital after an admission for epilepsy is largely unknown. In this study, we sought to quantify and compare readmission and emergency department (ED) presentations after hospitalisation for epilepsy in people with and without intellectual disability.

**Methods and findings:**

Using linked administrative datasets, we conducted a retrospective cohort study of people aged 5–64 years with an acute hospitalisation for epilepsy from 2005–2014 in New South Wales, Australia. Acute readmission and ED presentation rates within 30, 90, and 365 days of the index hospitalisation were estimated and compared between people with and without intellectual disability using modified Poisson regression. Of 13537 individuals with an index hospitalisation, 712 children and 1862 adults had intellectual disability. Readmission and ED presentation after the index hospitalisation were common in people with intellectual disability. Within 30 days, 11% of children and 15.6% of adults had an all-cause readmission and 18% of children and 23.5% of adults had an ED presentation. Over 60% of both children and adults presented to an ED within a year. Neurological, respiratory, and infectious conditions were overrepresented reasons for readmission in people with intellectual disability. Age-adjusted relative risks (RRs) within each period showed a higher risk of readmission and ED presentation in children and adults with intellectual disability than without. Most RRs remained statistically significant after controlling for covariates. The largest adjusted RRs were observed for readmission for epilepsy (RR 1.70, 95% CI: 1.42 to 2.04) and non-epilepsy related conditions (RR 1.73, 95%: CI 1.43 to 2.10) in children. Study limitations include lack of clinical data.

**Conclusions:**

Increased risk of returning to acute care after epilepsy hospitalisation suggests there is a need to improve epilepsy care for people with intellectual disability. We recommend research into strategies to improve management of both seizures and comorbidity.

## Introduction

Intellectual disability, defined by impairments in cognitive and adaptive functioning with onset during the developmental period [1, 2], affects approximately 1–2% of the population [3]. People with intellectual disability experience high rates of several health conditions including epilepsy [4]. A recent meta-analysis [5] found that 22% of people with intellectual disability have epilepsy, markedly higher than in the general population (<1.0%) [6].

Challenges in epilepsy management for people with intellectual disability include communication difficulties, high risk of refractory and frequent seizures, and neuropsychiatric comorbidities [7–10]. People with intellectual disability and epilepsy are at greater risk of hospitalisation and emergency department (ED) presentation than people with epilepsy or intellectual disability alone [11–14], and hospitalisation for epilepsy accounts for a large proportion of potentially avoidable hospitalisation for people with intellectual disability [15]. Individuals with both epilepsy and intellectual disability also experience other poor health outcomes, including increased risk of mortality [16].

Following hospitalisation for epilepsy, an important outcome is readmission. Repeated hospitalisations can induce psychological distress [17] and are costly [18]. They are thought to reflect suboptimal or poor continuity of care [19–21]. In the general population, the average unplanned (or acute) readmission rate within 30 days of epilepsy- or seizure-related hospitalisations is 10%, with epilepsy or seizure being the most common indication [20, 21]. Other common reasons for readmission within a year include psychiatric disorders [22, 23] and suicide attempts [24]. Few studies have reported readmission after epilepsy hospitalisation in people with intellectual disability, with one observing an increased risk of readmission in children with intellectual disability compared to children without [20]. However, the aforementioned study relied solely on hospital records to identify intellectual disability status, probably resulting in a sample with more severe disability or complex health needs [25]. Further research is needed to help understand how intellectual disability affects readmission risk after epilepsy hospitalisation.

This study aimed to compare rates of unplanned readmissions and ED presentations after hospitalisation for epilepsy among children and adults with and without intellectual disability. We also examined readmission for epilepsy and non-epilepsy conditions, specifically psychiatric disorders.

## Methods

### Data sources

We extracted data from an established linked dataset containing health and services records of people with neuropsychiatric disorders [25]. The data were drawn from multiple population-based administrative datasets from New South Wales (NSW), Australia as previously described [25]. Key datasets for this study were the Admitted Patient Data Collection (APDC), Emergency Department Data Collection (EDDC) and Registry of Births, Deaths and Marriages death records.

### Study population

Our study population was selected from the APDC, which contains all public and private hospital admissions in NSW. Principal and additional diagnoses are recorded for each admission using the International Statistical Classification of Diseases and Related Health Problems, Tenth Revision, Australian Modification (ICD-10-AM) [2].

**Fig 1:**
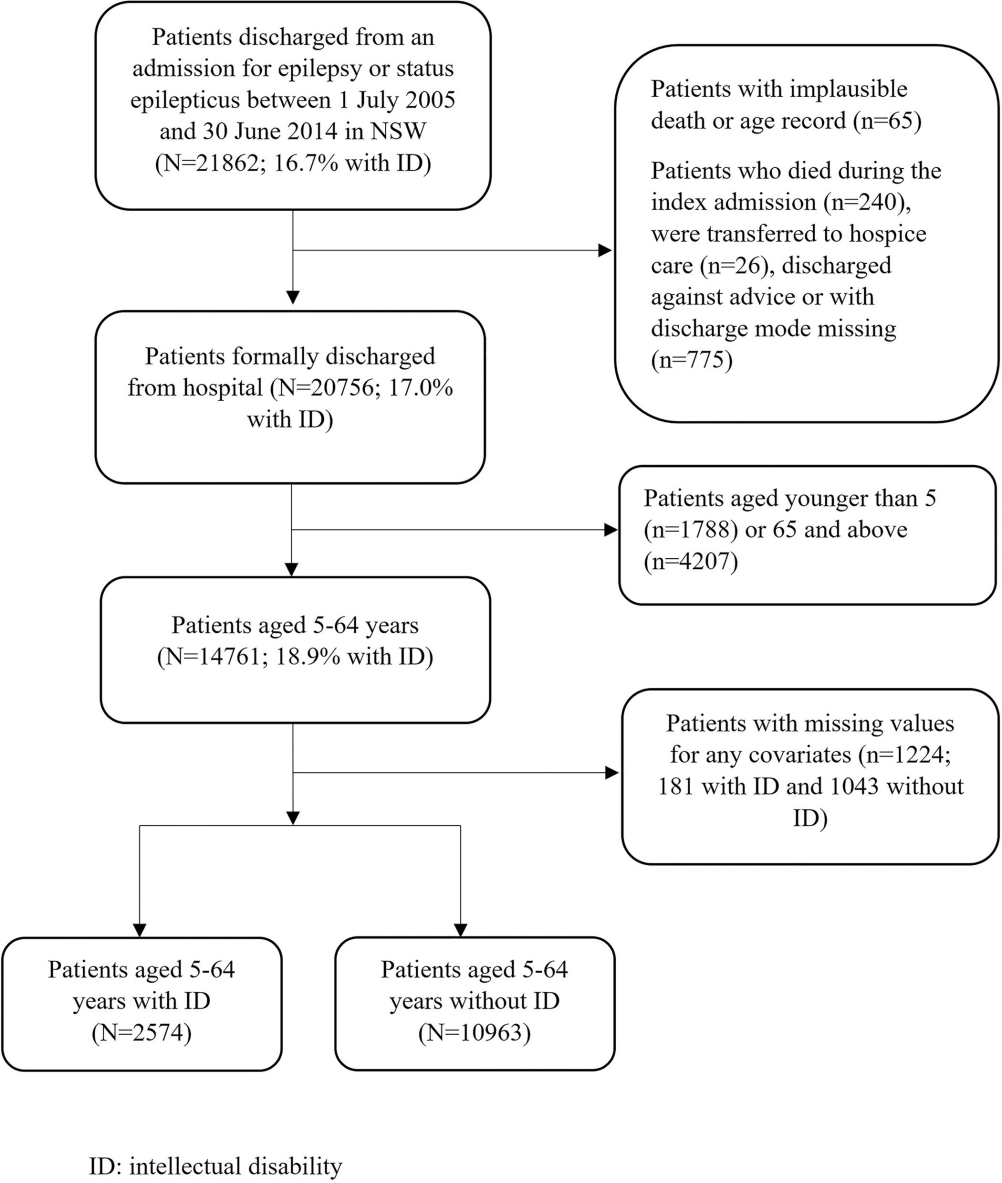
Cohort derivation flowchart.

Fig 1 describes how the cohort was derived. From all people who were discharged from hospital during the study period, we included people discharged from an unplanned acute care admission between 1 July 2005 and 30 June 2014 where the principal diagnosis recorded was epilepsy (G40) or status epilepticus (G41). S1 Table documents how we defined ‘unplanned’ and ‘acute’. The first admission was defined as the index admission. After excluding individuals with implausible death or age records, we excluded individuals if on discharge from the index admission they i) died, were transferred to hospice care, left hospital against advice, or had no discharge mode recorded [21]; ii) were younger than five or older than 64 years; or iii) had missing covariate data. The age limits were chosen to avoid bias introduced by young children yet to receive an intellectual disability diagnosis and older people for whom our data capture may be incomplete due to transition to aged care services [26]. Excluding older adults also reduced the likelihood of inclusion of epilepsy associated with late life disorders like dementia [27], which may complicate service use.

Diagnoses of intellectual disability were drawn from multiple health and disability datasets as previously described [25], by which people classified as having intellectual disability must have a diagnosis based on the Diagnostic and Statistical Manual of Mental Disorders IV or ICD-10 [1, 2]. This strategy resulted in formation of a cohort of people with intellectual disability who needed medical or disability service supports in NSW, representing about 1% of the NSW population in 2015 [25], comparable to the average estimate worldwide [3]. The remainder of the cohort was classified as not having intellectual disability.

We matched people with and without intellectual disability on age and sex using Coarsened Exact Matching to balance the distribution of covariates [28]. We formed strata defined by age group (2-years for children <16 years and 5-years for adults) and sex, with a weight applied to each individual’s observations to account for the number of people in their strata. This matching procedure retained all individuals in the analysis.

### Outcomes

We identified outcomes from the hospitalisation (APDC) and ED (EDDC) datasets. The EDDC contains information on ED presentations in 60% of NSW public hospitals between 1 July 2005 and 30 June 2016 [29]. The primary outcomes were all-cause unplanned hospital readmissions and ED presentations from the day of discharge from the index admission until day 30, day 90, day 365, or until death if that occurred first. Secondary outcomes were unplanned readmissions for epilepsy (G40-G41) and non-epilepsy conditions (G40 and G41 excluded), and specifically psychiatric disorders (F00-F69 and F80-F99), based on the principal diagnosis at discharge.

We did not include readmissions on the day of discharge from the index admission.

### Statistical analyses

All analyses were conducted separately for children (5–15 years) and adults (16–64 years) given the expected differences in the populations (e.g., some types of epilepsy), outcomes, and the age of transition from paediatric to adult inpatient services in NSW, Australia.

Descriptive statistics were used to compare demographic characteristics, index admission characteristics, and comorbidities for people with and without intellectual disability. We calculated the proportions of people with one or more readmissions or ED presentations during each follow-up period. The principal diagnosis of the first all-cause readmission was grouped by ICD-10-AM disease chapter. We also calculated the median number of days to the first outcome. T-test, Chi-square test (or Fisher exact test if ≤ five persons in one group), or Mann Whitney U test was used as applicable.

We fitted modified Poisson regression with robust estimation to examine associations between intellectual disability and the outcomes [30]; a survival analysis could not be used because the proportional hazards assumption was violated. We first fitted a model only adjusting for age at discharge from the index admission (not applicable for children) and then a model with select covariates derived from the APDC dataset. Age, sex, socio-economic status, index admission characteristics, and comorbidities are associated with intellectual disability or acute hospital visits after epilepsy hospitalisation [3, 21, 31]. Therefore, we selected age, sex, country of birth (Australia, overseas), Index of Relative Socio-economic Disadvantage (IRSD; based on area of residence), remoteness of residential area (Accessibility and Remoteness Index of Australia), hospitalisations in the previous year, characteristics of the index admission (length of stay, hospital type [public, private], mode of discharge [community, other accommodation or nursing home]), and comorbidity status as covariates. For comorbidity status, we retrieved all diagnoses recorded at the index admission and admissions in the previous year to obtain the Charlson comorbidity index (CCI) [32] and psychiatric diagnoses. The latter excluded intellectual disability and dementia. The CCI and psychiatric disorders were not included as covariates in the modelling for children due to the low prevalence of these conditions in this age group. The most common neuropsychiatric disorders in children with intellectual disability are other neurodevelopmental conditions (e.g., autism and attention deficit hyperactivity disorder) [33], which are less common in the general population [34] and, more importantly, may lie on the causal pathway.

In the main analysis, we excluded individuals with missing data (less than 10%), as the cohort characteristics before and after the exclusion were comparable. We also performed a sensitivity analysis to test the impact of excluding people with missing data. The level of significance was set to p<0.05. We performed data matching and analyses in Stata 15.

### Ethics approval

This study was approved by NSW Population & Health Services Research Ethics Committee, and access to the data sets was granted by relevant data custodians. As the research met specific safeguards, the requirement for informed consent was waived by the presiding ethics committee.

## Results

### Cohort characteristics

Out of 21862 patients with an admission for epilepsy during the study period, 20756 patients were formally discharged from the hospital, among which, we included all patients aged 5–64 years without missing records for any covariates (2574 with intellectual disability; Fig 1). This final cohort consisted of 2104 children and 11433 adults. Children had a higher prevalence of intellectual disability than adults (33.8% versus 16.3%; Table 1). Cohort characteristics and outcomes before and after excluding participants with missing data showed that missingness was unrelated to the outcomes (characteristics of participants with complete data shown in S2 Table).

**Table 1:** Cohort characteristics at the index admission for children and adults with and without Intellectual disability (ID) (n, %).

	Children (N = 2,104)			Adults (N = 11,433)		
	ID (N = 712)	Non-ID (N = 1,392)	P value^c^	ID (N = 1,862)	Non-ID (N = 9,571)	P value^c^
**Male**	409 (57.4)	743 (53.4)	0.076	1064 (57.1)	5488 (57.3)	0.875
**Median (IQR) age (years)**	9.5 (7–12)	10.8 (8–11)		36.7 (25–49)	39.8 (28–51)	
**Age range (years)**						
16–24	N/A	N/A		469 (25.2)	1754 (18.3)	<0.001
25–44	N/A	N/A		786 (42.2)	4166 (43.5)	
45–64	N/A	N/A		607 (32.6)	3651 (38.2)	
**Born in Australia**	674 (94.7)	1289 (92.6)	0.073	1735 (93.2)	7743 (80.9)	<0.001
**Remoteness of residence**						
Major city	469 (65.9)	939 (67.5)	0.348	1225 (65.8)	6419 (67.1)	0.389
Inner regional	165 (23.2)	328 (23.6)		442 (23.7)	2240 (23.4)	
Outer regional/remote/very remote	78 (11.0)	125 (9.0)		195 (10.5)	912 (9.5)	
**Index of relative socio-economic disadvantage based on place of residence**						
1–2 (most disadvantaged)	143 (20.1)	366 (26.3)	<0.001	366 (19.7)	2390 (25.0)	<0.001
3–4	148 (20.8)	280 (20.1)		284 (20.6)	2107 (22.0)	
5–6	161 (22.6)	278 (20.0)		409 (22.0)	1865 (19.5)	
7–8	150 (21.1)	204 (14.7)		417 (22.4)	1590 (16.6)	
9–10 (least disadvantaged)	110 (15.5)	264 (19.0)		286 (15.4)	1619 (16.9)	
**Characteristics of the index admission**						
** Private health insurance**	179 (25.1)	419 (30.1)	0.017	205 (11.0)	2020 (21.1)	<0.001
** Public hospital**	>700 (>95)^a^	1374 (98.7)	0.024	1853 (99.5)	9485 (99.1)	0.071
** Median (IQR) length of stay (days)**	1.0 (1–2)	1.0 (1–2)		1.0 (1–3)	1.0 (1–3)	
** Length of stay (days)**						
1–2	542 (76.1)	1186 (85.2)	<0.001	1284 (69.0)	7098 (74.2)	<0.001
3–6	120 (16.9)	176 (12.6)		383 (20.6)	1617 (16.9)	
≥7	50 (7.0)	30 (2.2)		195 (10.5)	856 (8.9)	
** Mode of separation**						
Discharge by hospital	>700 (>95)^a^	>1350 (>95)^a^	1.000	1828 (98.2)	9496 (99.2)	<0.001
Transfer to nursing home	N/A	N/A		18 (1.0)	58 (0.6)	
Transfer to other accommodation	<5^a^	<5^a^		16 (0.9)	17 (0.2)	
**≥ one admission in the year prior**	369 (51.8)	399 (28.7)	<0.001	897 (48.2)	4215 (44.0)	0.001
**Charlson Comorbidity Index**						
0	671 (94.2)	1338 (96.1)	0.126	1613 (86.6)	7990 (83.5)	<0.001
1–2	35 (4.9)	48 (3.5)		190 (10.2)	950 (9.9)	
≥3	6 (0.8)	6 (0.4)		59 (3.2)	631 (6.6)	
Psychiatric comorbidity^b^	269 (37.8)	82 (5.9)	<0.001	536 (28.8)	2697 (28.2)	0.590

^a^ True value and/or percentage were censored to ensure confidentiality.

^b^ ICD-10 codes for psychiatric comorbidity: F00-F99, except for intellectual disability (F70-F79) and dementia (F00-F03 or F05.1; according to the codes included in the Charlson Comorbidity Index).

^c^ P value was estimated based on independent t-test for continuous variables and Chi-squared test for categorical variables.

Compared to adults without intellectual disability, a higher proportion of adults with intellectual disability were born in Australia (P<0.001). Individuals with intellectual disability, regardless of age, were more likely to live in moderately socioeconomically disadvantaged areas (P<0.001).

At the index admission, almost all people attended a public hospital and were discharged to the community. Therefore, we did not include the hospital and discharge type variable in the regression model in the next stage. Compared to people without intellectual disability, people with intellectual disability were less likely to hold private health insurance and more likely to have longer hospital stays (≥3 days) and an admission in the previous year (P≤0.001). Compared to adults without intellectual disability, adults with intellectual disability were slightly more likely to have a CCI of 0. Psychiatric comorbidities were common in all adults (28.8% and 28.2% for those with and without intellectual disability, respectively) and children with intellectual disability (37.8%), but not in children without (5.9%). As noted in the statistical analyses section, the difference in prevalence of psychiatric comorbidities in children with and without intellectual disability may primarily be due to the higher risk of neurodevelopmental disorders in children with intellectual disability. Neurodevelopmental conditions (e.g. autism and attention deficit hyperactivity disorder) are typically diagnosed early in life, whereas other mental health conditions (e.g. depression and anxiety) are more likely to be diagnosed during young adulthood [35].

### All-cause readmissions

Compared to people without intellectual disability, a larger proportion of people with intellectual disability were readmitted to hospital (Table 2). Seventy-eight (11.0%) children and 291 (15.6%) adults with intellectual disability had at least one readmission within 30 days. The proportions increased to 46.4% (children) and 52.8% (adults) within a year. Almost one third of people with intellectual disability had more than one all-cause readmission within a year, compared to 9.4% of children and 20.7% of adult comparators. There was no difference in the number of days to the first readmission between people with and without intellectual disability (Table 3).

**Table 2:** Comparison of the proportions of people with and without intellectual disability with at least one outcome within each follow-up period by age.

Outcomes	Children				Adults			
	n (%)		RR (95% CI)		n (%)		RR (95% CI)	
	ID	Non-ID	Model 1^a^	Model 2^b^	ID	Non-ID	Model 1^a^	Model 2^b^
**All-cause readmission**								
Within 30 days	78 (11.0)	98 (7.0)	1.65 (1.23–2.21)	1.26 (0.94–1.69)	291 (15.6)	1011 (10.6)	1.51 (1.34–1.70)	1.42 (1.26–1.61)
Within 90 days	157 (22.1)	191 (13.7)	1.75 (1.44–2.14)	1.42 (1.15–1.74)	506 (27.2)	1974 (20.6)	1.35 (1.24–1.47)	1.27 (1.17–1.39)
Within 365 days	328 (46.1)	376 (27.0)	1.81 (1.60–2.05)	1.58 (1.39–1.79)	983 (52.8)	3907 (40.8)	1.33 (1.26–1.40)	1.26 (1.19–1.32)
**Readmission for epilepsy**								
Within 30 days	49 (6.9)	64 (4.6)	1.57 (1.09–2.28)	1.25 (0.86–1.81)	143 (7.7)	419 (4.4)	1.71 (1.42–2.06)	1.64 (1.36–1.97)
Within 90 days	90 (12.6)	118 (8.5)	1.63 (1.25–2.13)	1.43 (1.08–1.88)	267 (14.3)	869 (9.1)	1.55 (1.36–1.76)	1.47 (1.29–1.68)
Within 365 days	209 (29.4)	230 (16.5)	1.90 (1.60–2.25)	1.70 (1.42–2.04)	572 (30.7)	1907 (19.9)	1.53 (1.41–1.66)	1.44 (1.33–1.56)
**Readmission for non-epilepsy conditions**								
Within 30 days	34 (4.8)	38 (2.7)	1.98 (1.24–3.18)	1.36 (0.84–2.18)	170 (9.1)	643 (6.7)	1.42 (1.21–1.68)	1.33 (1.13–1.57)
Within 90 days	90 (12.6)	89 (6.4)	2.18 (1.63–2.92)	1.54 (1.14–2.08)	305 (16.4)	1302 (13.6)	1.27 (1.14–1.43)	1.20 (1.07–1.35)
Within 365 days	200 (28.1)	192 (13.8)	2.21 (1.84–2.66)	1.73 (1.43–2.10)	651 (35.0)	2827 (29.5)	1.24 (1.16–1.33)	1.16 (1.09–1.25)
**Readmission for psychiatric disorders**								
Within 30 days	<5^c^	5 (0.4)	N/A^d^	N/A^d^	40 (2.2)	140 (1.5)	1.52 (1.07–2.16)	1.47 (1.03–2.10)
Within 90 days	7 (1.0)	9 (0.7)	N/A^d^	N/A^d^	76 (4.1)	291 (3.0)	1.41 (1.10–1.80)	1.35 (1.05–1.73)
Within 365 days	12 (1.7)	22 (1.6)	N/A^d^	N/A^d^	161 (8.7)	695 (7.3)	1.23 (1.04–1.44)	1.14 (0.97–1.33)
**ED presentation**								
Within 30 days	128 (18.0)	214 (15.4)	1.23 (1.00–1.51)	1.03 (0.84–1.27)	438 (23.5)	1628 (17.0)	1.37 (1.25–1.50)	1.29 (1.18–1.42)
Within 90 days	241 (33.9)	394 (28.3)	1.26 (1.10–1.45)	1.08 (0.94–1.25)	758 (40.7)	3012 (31.5)	1.29 (1.21–1.37)	1.21 (1.14–1.29)
Within 365 days	456 (64.0)	697 (50.1)	1.31 (1.22–1.42)	1.23 (1.13–1.33)	1264 (67.9)	5477 (57.2)	1.19 (1.15–1.23)	1.14 (1.10–1.18)

^a^ Model 1 was adjusted for age for adults and unadjusted for children.

^b^ Model 2 was adjusted for age, sex, country of birth, residential remoteness, IRSD, private health insurance at index admission, length of stay at index admission, Charlson comorbidity index, comorbid psychiatric disorders and admission during the last year for adults; age, Charlson Comorbidity Index, and psychiatric comorbidities were excluded from the model for children.

^c^ True value and percentage were censored to ensure confidentiality.

^d^ N/A: Readmissions for psychiatric disorders are not applicable to children.

**Table 3:** Days to the first outcome (median, IQR) within each follow-up period for people with and without intellectual disability with at least one outcome.

Outcomes		Children					Adults			
		ID	Non-ID				ID	Non-ID		
	n^a^	Median (IQR)	n^a^	Median (IQR)	P value^c^	n^a^	Median (IQR)	n^a^	Median (IQR)	P value^c^
**All-cause readmissions**										
Within 30 days	78	9 (4–21)	98	9 (4–18)	0.695	291	9 (4–19)	1011	11 (4–20)	0.037
Within 90 days	157	31 (9–59)	191	29 (8–53)	0.622	506	25 (7–51)	1974	30 (11–55)	0.003
Within 365 days	328	97 (33–203)	376	90 (28–205)	0.363	983	83 (24–194)	3907	88 (29–193)	0.149
**Readmission for epilepsy**										
Within 30 days	49	12 (4–23)	64	12 (4–23)	0.451	143	9 (3–30)	419	11 (4–20)	0.778
Within 90 days	90	27 (12–51)	118	27 (12–51)	0.844	267	28 (8–55)	869	32 (11–58)	0.128
Within 365 days	209	113 (34–223)	230	113 (34–223)	0.056	572	101 (31–210)	1907	106 (37–204)	0.517
**Readmission for non-epilepsy conditions**										
Within 30 days	34	8 (5–17)	38	9.5 (4–15)	0.964	170	10 (4–18)	643	11 (5–21)	0.077
Within 90 days	90	43.5 (14–68)	89	37 (11–56)	0.214	305	26 (9–51)	1302	31 (12–57)	0.011
Within 365 days	200	106 (49–220.5)	192	104.5 (38–217)	0.466	651	103 (28–212)	2827	106 (35–211)	0.228
**Readmission for psychiatric disorders**										
Within 30 days	<5^b^	15 (4–17)	5	15 (7–18)	0.764	40	11 (5–21)	140	13 (7–23)	0.336
Within 90 days	7	56 (15–77)	9	22 (15–53)	0.340	76	29 (10–49)	291	32 (14–57)	0.163
Within 365 days	12	79 (37–225)	22	114 (52–237)	0.829	161	94 (31–212)	695	113 (40–227)	0.160
**ED presentation**										
Within 30 days	128	8 (3–18)	214	8 (4–17)	0.965	438	8 (3–16)	1628	9 (3–18)	0.268
Within 90 days	241	27 (8–54)	394	25 (8–53)	0.765	758	24 (7–48)	3012	27 (8–53)	0.018
Within 365 days	456	81 (26–182)	697	73 (21–175)	0.208	1264	61 (15–145)	5477	75 (23–177)	<0.001

^a^ The number of patients having at least one outcome (readmission or ED presentation).

^b^ True value was censored to ensure confidentiality.

^c^ P value was estimated based on Mann Whitney U.

**Fig 2:**
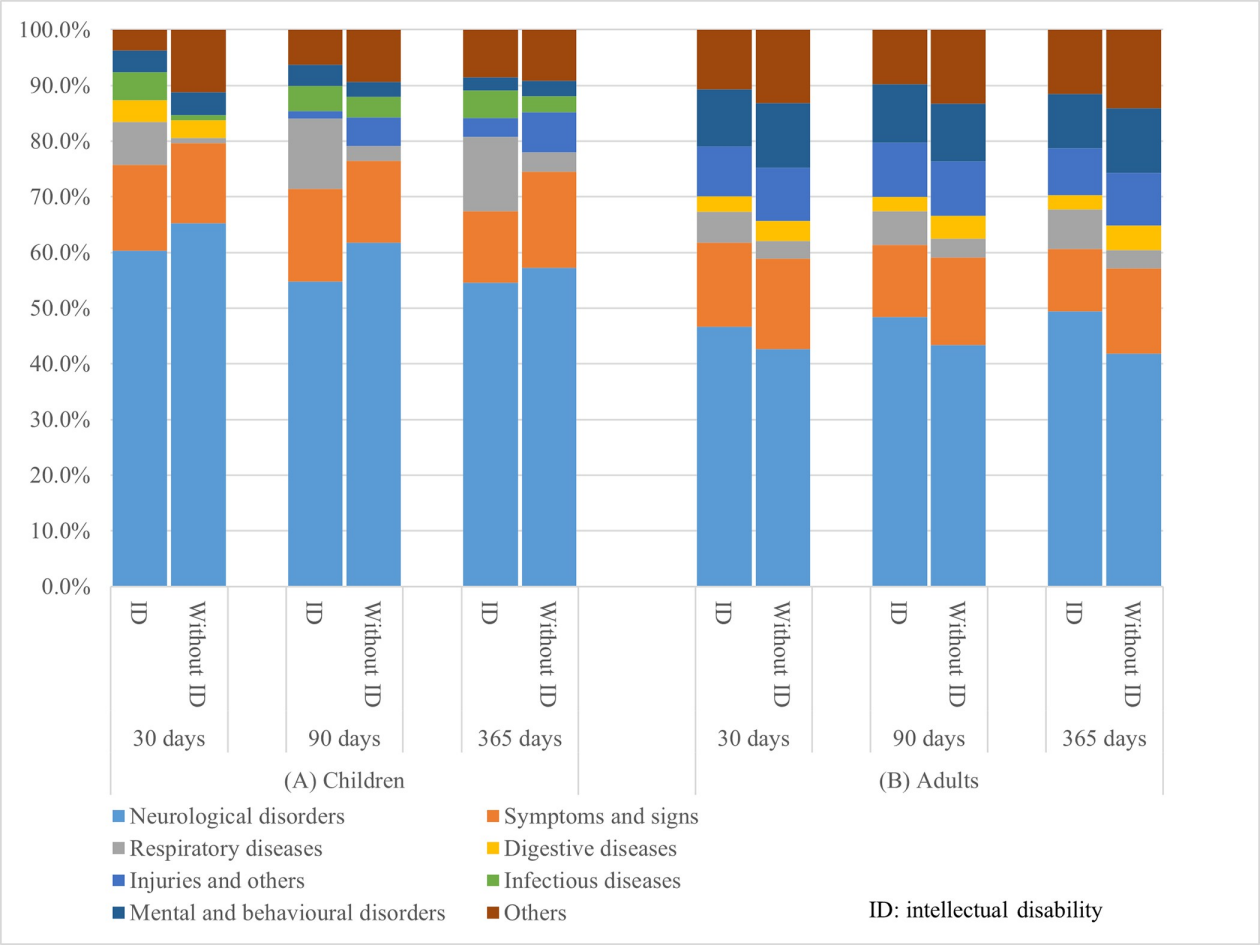
The six most frequent primary diagnoses at the first readmission within each follow-up period for children (A) and adults (B) by intellectual disability status.

The dominant reason for the first readmission in people with and without intellectual disability was neurological disorders (>95% of which were epilepsy), followed by unspecified symptoms (Fig 2). Neurological conditions were slightly over-represented in adults with intellectual disability within each follow-up period. There was a larger proportion of readmissions for respiratory conditions in adults and children with intellectual disability (particularly lung diseases due to external agents, influenza, and pneumonia; the latter two were only overrepresented in children), and infectious diseases in children with intellectual disability, than those without.

The age-adjusted model showed a higher risk of all-cause readmission in people with intellectual disability compared to people without intellectual disability within each follow-up period. After adjusting for other covariates, the increased risk remained significant for all except 30-day readmission in children. The highest relative risk was found for children within 365 days (RR 1.58, 95%CI: 1.39 to 1.79).

### Readmissions for specific reasons

Compared to people without intellectual disability, higher proportions of people with intellectual disability were readmitted for both epilepsy and non-epilepsy conditions across the follow-up periods, but the median times to readmission were comparable (Tables 2 and 3). Psychiatric readmission rates were generally low and similar between groups.

The age-adjusted model showed a higher risk of epilepsy and non-epilepsy readmission in people with intellectual disability than those without in each follow-up period. In the multivariable model, the increased risk remained significant for all except 30-day epilepsy or non-epilepsy readmission in children. The highest risk was observed for epilepsy and non-epilepsy readmission in children within a year (RR 1.70, 95% CI: 1.42 to 2.04; RR 1.73, 95% CI: 1.43 to 2.10, respectively).

The age-adjusted model also showed an increased risk of psychiatric readmission within each follow-up period in adults with intellectual disability compared to those without (Table 2), which remained significant for readmissions within 30 and 90 days after adjusting for covariates. The largest RR was observed within 30 days of the index admission (RR 1.47, 95% CI: 1.03 to 2.10).

### ED presentations

In both observation periods (30 and 365 days), a greater proportion of people with intellectual disability presented to ED compared to those without (Table 2). Almost half the children and adults with intellectual disability had more than one ED presentation within 365 days, versus around one-third of the comparison groups. Within 365 days, the median number of days to the first ED presentation was shorter for adults with intellectual disability than those without (Table 3).

The age-adjusted RR showed a significantly increased risk of ED presentations in adults with intellectual disability within all follow-up periods and in children with intellectual disability within 90 and 365 days (Table 2). After adjusting for covariates, all RRs remained significant for adults and only the RR within 365 days remained significant for children. The largest RR was observed for adults within 30 days (RR 1.29, 95% CI: 1.18 to 1.42).

## Discussion

In this population-based study using linked administrative datasets, we showed re-entry to acute care after epilepsy hospitalisation was common, especially in adults with intellectual disability. Intellectual disability status was associated with a higher readmission and ED presentation risk within 30, 90, and 365 days of the index hospitalisation. The risk of readmission for both epilepsy and non-epilepsy conditions was elevated. Over-represented causes of readmission included neurological and respiratory conditions in all people with intellectual disability, and infectious disorders in children with intellectual disability. These findings suggest there is a need to provide specialised and interdisciplinary support for inpatient and post-acute care support around epilepsy management for people with intellectual disability.

Our study confirmed prior evidence showing a high rate of 30-day all-cause readmission in children with intellectual disability following epilepsy hospitalisation [20]. Expanding the previous knowledge, we further showed high readmission and ED presentation rates within 30, 90, and 365 days in both children and adults with intellectual disability. The proportion of people readmitted was higher in adults than children with intellectual disability, resembling the trend in the general population [31]. We also found a high rate of repeated ED presentations within a year in people with intellectual disability, possibly indicating inadequate acute seizure management. Our findings highlight the need to address the determinants of frequent ED use in people with epilepsy and intellectual disability. Collectively, these results show a high rate of acute care utilisation after discharge from epilepsy hospitalisation among people with intellectual disability.

Intellectual disability was associated with increased risk of readmission, for both epilepsy and non-epilepsy conditions, and ED presentation within the short- and long-term in adults and within the long-term in children. We observed no association between 30-day all-cause readmission and intellectual disability status in children, contrasting with the 50% excess readmission risk reported in an American cohort (1–17 years) using a hospital register [20]. The discordant finding may reflect differences in health systems between two countries, or may have occurred due to methodological differences. We included only children aged 5–15 years, adjusted for previous hospitalisations as a potential indicator of general health status, and identified intellectual disability from multiple reliable sources.

The association between higher risk of acute care and intellectual disability was independent of sociodemographic, health, and index admission characteristics in this study. We surmise that factors that have not been controlled for, including epilepsy characteristics and potentially inappropriate or inadequate management, may have driven the excess acute care in people with intellectual disability. More severe and frequent seizures often occurring with intellectual disability could result in more inpatient care, which may be entirely appropriate. However, previous research has revealed multiple gaps in epilepsy care for people with intellectual disability, including a low level of multidisciplinary care [36] and inadequate use of evidence-based treatments [37]. Bridging these gaps by introducing specialised services with expertise in intellectual disability and epilepsy, while mitigating the general barriers people with intellectual disability experience in seeking quality health care [38, 39], has the potential to reduce seizure frequency and improve the comorbidity management, thus reducing the need for recurrent emergency hospital care.

We observed variations in relative risks of readmission over time since the index hospitalisation between children and adults. Generally, the disparity in readmission rates between those with and without intellectual disability was the largest within 30 days and decreased over time in adults, while it was the lowest within 30 days and peaked within one year in children. As 30-day all-cause readmission is widely used to indicate the quality of inpatient care, and one-year readmission might be partly attributed to outpatient care after discharge, this finding may suggest care gaps are more related to inpatient care for adults and post-discharge care for children.

Epilepsy or seizure, the leading reason for readmission in the general population with epilepsy [20, 21], accounted for an even larger proportion of readmissions in people with intellectual disability. This suggests that there is a need for improved clinical epilepsy care pathways in this high-risk patient group, which might emphasise continuity of care in the community, more frequent reviews of management, and access to highly specialised reviews for those with complex epilepsy and health needs. Disability sector staff could also be better equipped to support seizure management, including timely access to appropriate health experts. We also observed an overrepresentation of readmissions for non-epilepsy conditions in people with intellectual disability. Hospitalisation for respiratory disorders, specifically influenza, pneumonia, and lung diseases due to external agents, is consistent with evidence of more readmissions for respiratory diseases in people with intellectual disability [40, 41]. This suggests a need for better influenza and aspiration prevention in their epilepsy care. The excess readmission for infectious diseases within 30 days in children was unexpected, which may reflect more undetected underlying central nervous system infection [42]. We did not observe more readmissions for injuries in people with intellectual disability despite evidence of an association between injuries and intellectual disability from a Scottish cohort [43], suggesting injuries in our cohort were mainly related to epilepsy rather than disability.

Consistent with prior evidence of an association between neuropsychiatric disorders and intellectual disability in people with epilepsy [10], we found an association between intellectual disability and psychiatric readmission risk within 30 and 90 days in adults. The association was most pronounced within 30 days, suggesting the need to improve screening and enhance monitoring for neuropsychiatric symptoms during and shortly after epilepsy hospitalisations, respectively, in adults with intellectual disability.

We used population-based databases to build one of the largest intellectual disability cohorts internationally. Combining health and disability registers enabled a more representative cohort than relying on one register alone [20], thus yielding more generalisable results. The data linkage allowed us to ascertain outcomes systematically and consistently for all participants and to account for death. Study limitations include potential coding and recording errors and lack of clinical information in the administrative datasets, particularly the severity of epilepsy and intellectual disability. While we were not able to ascertain disability severity, we have generated evidence on the needs of people with intellectual disability as a whole and identified areas that could benefit from further research. Furthermore, as the diagnosis of epilepsy not otherwise specified was common, we were unable to explore variation by epilepsy subtype. Additionally, although the residential setting could influence ED visits in people with intellectual disability [44], we did not have complete data on the residential setting or living arrangements. Finally, while the participating EDs are large centres that serve a substantial proportion of the NSW population, the lack of population coverage of ED presentations means that we have underestimated ED presentations [29].

In conclusion, our findings provide evidence of excess repeat acute care in both children and adults with intellectual disability and epilepsy in a universal healthcare setting. They suggest the need for interdisciplinary team management in the care of people with intellectual disability and epilepsy in both hospital and community settings. We recommend further research on strategies to improve management of seizures and comorbidities in people with intellectual disability and epilepsy. Future research should also identify drivers of repeat acute care, particularly preventable factors, and how they differ between people with and without intellectual disability. As a result of continuous deinstitutionalisation, people with epilepsy and intellectual disability increasingly attend mainstream healthcare services. Failure to understand and cater to their needs may inadvertently widen the health disparities experienced by people with intellectual disability.

## Supporting information

S1 Table Variables and respective values used to define an unplanned hospitalisation or readmission. (DOCX)

S2 Table Baseline characteristics of children and adults, including those with incomplete data, with and without intellectual disability (n, %). (DOCX)

## References

[1] American Psychiatric Association. Diagnostic and statistical manual of mental disorders. 4th ed: Washington, DC: American Psychiatric Association; 1994.

[2] World Health Organization. ICD-10: international statistical classification of diseases and related health problems: tenth revision. 2nd ed ed. Geneva: World Health Organization; 2004.

[3] Maulik PK, Mascarenhas MN, Mathers CD, Dua T, Saxena S. Prevalence of intellectual disability: A meta-analysis of population-based studies. *Res Dev Disabil*. 2011;32(2):419–36. doi: 10.1016/j.ridd.2010.12.018 21236634

[4] Carey IM, Shah SM, Hosking FJ, DeWilde S, Harris T, Beighton C, et al. Health characteristics and consultation patterns of people with intellectual disability: a cross-sectional database study in English general practice. *Br J Gen Pract*. 2016;66(645):e264–70. doi: 10.3399/bjgp16X684301 26906630 PMC4809710

[5] Robertson J, Hatton C, Emerson E, Baines S. Prevalence of epilepsy among people with intellectual disabilities: A systematic review. *Seizure*. 2015;29:46–62. doi: 10.1016/j.seizure.2015.03.016 26076844

[6] Fiest KM, Sauro KM, Wiebe S, Patten SB, Kwon CS, Dykeman J, et al. Prevalence and incidence of epilepsy: A systematic review and meta-analysis of international studies. *Neurology*. 2017;88(3):296–303. doi: 10.1212/WNL.0000000000003509 27986877 PMC5272794

[7] Kalilani L, Sun X, Pelgrims B, Noack-Rink M, Villanueva V. The epidemiology of drug-resistant epilepsy: A systematic review and meta-analysis. *Epilepsia*. 2018;59(12):2179–93. doi: 10.1111/epi.14596 30426482

[8] McGrother CW, Bhaumik S, Thorp CF, Hauck A, Branford D, Watson JM. Epilepsy in adults with intellectual disabilities: Prevalence, associations and service implications. *Seizure*. 2006;15(6):376–86. doi: 10.1016/j.seizure.2006.04.002 16782360

[9] Branford D, Bhaumik S, Duncan F. Epilepsy in adults with learning disabilities. *Seizure*. 1998;7(6):473–7. doi: 10.1016/s1059-1311(98)80005-8 9888491

[10] van Ool JS, Snoeijen-Schouwenaars FM, Schelhaas HJ, Tan IY, Aldenkamp AP, Hendriksen JGM. A systematic review of neuropsychiatric comorbidities in patients with both epilepsy and intellectual disability. *Epilepsy Behav*. 2016;60:130–7. doi: 10.1016/j.yebeh.2016.04.018 27206231

[11] Morgan CL, Baxter H, Kerr MP. Prevalence of epilepsy and associated health service utilization and mortality among patients with intellectual disability. *Am J Ment Retard*. 2003;108(5):293–300. doi: 10.1352/0895-8017(2003)108&lt;293:POEAAH&gt;2.0.CO;2 12901705

[12] Puka K, Smith ML, Moineddin R, Snead OC, Widjaja E. Health resource utilization varies by comorbidities in children with epilepsy. *Epilepsy Behav*. 2016;57(Pt A):151–4. doi: 10.1016/j.yebeh.2016.02.011 26953845

[13] Balogh R, Brownell M, Ouellette-Kuntz H, Colantonio A. Hospitalisation rates for ambulatory care sensitive conditions for persons with and without an intellectual disability-a population perspective. *J Intellect Disabil Res*. 2010;54(9):820–32. doi: 10.1111/j.1365-2788.2010.01311.x 20704636

[14] Lindgren S, Lauer E, Momany E, Cope T, Royer J, Cogan L, et al. Disability, hospital care, and cost: utilization of emergency and inpatient care by a cohort of children with intellectual and developmental disabilities. *J Pediatr*. 2020. doi: 10.1016/j.jpeds.2020.08.084 32890584 PMC7885996

[15] Hosking FJ, Carey IM, DeWilde S, Harris T, Beighton C, Cook DG. Preventable emergency hospital admissions among adults with intellectual disability in England. *Ann Fam Med*. 2017;15(5):462–70. doi: 10.1370/afm.2104 28893817 PMC5593730

[16] Robertson J, Hatton C, Emerson E, Baines S. Mortality in people with intellectual disabilities and epilepsy: A systematic review. *Seizure*. 2015;29:123–33. doi: 10.1016/j.seizure.2015.04.004 26076855

[17] Mengoni SE, Gates B, Parkes G, Wellsted D, Barton G, Ring H, et al. "Sometimes, it just stops me from doing anything": A qualitative exploration of epilepsy management in people with intellectual disabilities and their carers. *Epilepsy Behav*. 2016;64(Pt A):133–9. doi: 10.1016/j.yebeh.2016.09.029 27736660 PMC5140003

[18] Bellon ML, Barton C, McCaffrey N, Parker D, Hutchinson C. Seizure-related hospital admissions, readmissions and costs: comparisons with asthma and diabetes in South Australia. *Seizure*. 2017;50:73–9. doi: 10.1016/j.seizure.2017.06.005 28624716

[19] Terman SW, Guterman EL, Hill CE, Betjemann JP, Burke JF. Factors associated with 30-day readmission for patients hospitalized for seizures. *Neurol Clin Pract*. 2020;10(2):122–30. doi: 10.1212/CPJ.0000000000000688 32309030 PMC7156187

[20] Kessler SK, Blank LJ, Glusman J, Thibault D, Massey S, Abend NS, et al. Unplanned readmissions of children with epilepsy in the United States. *Pediatr Neurol*. 2020;108:93–8. doi: 10.1016/j.pediatrneurol.2020.01.010 32299746 PMC13075384

[21] Blank LJ, Crispo JAG, Thibault DP, Davis KA, Litt B, Willis AW. Readmission after seizure discharge in a nationally representative sample. *Neurology*. 2018;92(5):e429–42. doi: 10.1212/wnl.0000000000006746 30578373 PMC6369906

[22] Kim AM, Rossi KC, Jetté N, Yoo JY, Hung K, Dhamoon MS. Increased risk of hospital admission for mood disorders following admission for epilepsy. *Neurology*. 2018;91(9):e800–e10. doi: 10.1212/01.wnl.0000542492.00605.9d 30068636

[23] Rossi KC, Kim AM, Jetté N, Yoo JY, Hung K, Dhamoon MS. Increased risk of hospital admission for ICD-9-CM psychotic episodes following admission for epilepsy. *Epilepsia*. 2018;59(8):1603–11. doi: 10.1111/epi.14508 29974458

[24] Xu KY, Rossi KC, Kim AM, Jetté N, Yoo JY, Hung K, et al. Risk of readmission for suicide attempt after epilepsy hospitalization. *Epilepsy Behav*. 2018;83:124–30. doi: 10.1016/j.yebeh.2018.03.037 29702413

[25] Reppermund S, Heintze T, Srasuebkul P, Reeve R, Dean K, Smith M, et al. Health and wellbeing of people with intellectual disability in New South Wales, Australia: a data linkage cohort. *BMJ open*. 2019;9(9):e031624. doi: 10.1136/bmjopen-2019-031624 31575581 PMC6773320

[26] Florio T, Trollor J. Mortality among a cohort of persons with an intellectual disability in New South Wales, Australia. *J Appl Res Intellect Disabil*. 2015;28(5):383–93. doi: 10.1111/jar.12190 25994286

[27] McCarron M, O’Dwyer M, Burke E, McGlinchey E, McCallion P. Epidemiology of Epilepsy in Older Adults With an Intellectual Disability in Ireland: Associations and Service Implications. *Am J Intellect Dev Disabil*. 2014;119(3):253–60. doi: 10.1352/1944-7558-119.3.253 24871793

[28] Iacus SM, King G, Porro G. Causal inference without balance checking: Coarsened exact matching. *Polit Anal*. 2012;20(1):1–24.

[29] The Centre for Health Record Linkage (CHeReL). Data dictionary. Updated March 2020. Available rom: https://www.cherel.org.au/data-dictionaries#section5.

[30] Zou G. A modified poisson regression approach to prospective studies with binary data. *Am J Epidemiol*. 2004;159(7):702–6. doi: 10.1093/aje/kwh090 15033648

[31] Mitchell RJ, Herkes G, Nikpour A, Bleasel A, Shih P, Vagholkar S, et al. Examining health service utilization, hospital treatment cost, and mortality of individuals with epilepsy and status epilepticus in New South Wales, Australia 2012–2016. *Epilepsy Behav*. 2018;79:9–16. doi: 10.1016/j.yebeh.2017.11.022 29223007

[32] Quan H, Li B, Couris CM, Fushimi K, Graham P, Hider P, et al. Updating and validating the Charlson comorbidity index and score for risk adjustment in hospital discharge abstracts using data from 6 countries. *Am J Epidemiol*. 2011;173(6):676–82. doi: 10.1093/aje/kwq433 21330339

[33] Buckley N, Glasson EJ, Chen W, Epstein A, Leonard H, Skoss R, et al. Prevalence estimates of mental health problems in children and adolescents with intellectual disability: A systematic review and meta-analysis. *Aust N Z J Psychiatry*. 2020;54(10):970–84. doi: 10.1177/0004867420924101 32475125

[34] Davies S, Heyman I, Goodman R. A population survey of mental health problems in children with epilepsy. *Dev Med Child Neurol*. 2003;45(5):292–5. doi: 10.1017/s0012162203000550 12729141

[35] Solmi M, Radua J, Olivola M, Croce E, Soardo L, Salazar de Pablo G, et al. Age at onset of mental disorders worldwide: large-scale meta-analysis of 192 epidemiological studies. *Mol Psychiatry*. 2022;27(1):281–95. doi: 10.1038/s41380-021-01161-7 34079068 PMC8960395

[36] Liew A. An audit of the management of epilepsy in children and young people with intellectual disabilities. *J Neurol Neurosurg Psychiatry*. 2015;86(9):e3–e. doi: 10.1136/jnnp-2015-311750.52

[37] Thompson R, Linehan C, Glynn M, Kerr MP. A qualitative study of carers’ and professionals’ views on the management of people with intellectual disability and epilepsy: a neglected population. *Epilepsy Behav*. 2013;28(3):379–85. doi: 10.1016/j.yebeh.2013.05.024 23856071

[38] Weise J, Pollack A, Britt H, Trollor JN. Primary health care for people with an intellectual disability: an exploration of consultations, problems identified, and their management in Australia. *J Intellect Disabil Res*. 2017;61(5):399–410. doi: 10.1111/jir.12352 28116807

[39] Iacono T, Bigby C, Unsworth C, Douglas J, Fitzpatrick P. A systematic review of hospital experiences of people with intellectual disability. *BMC Health Serv Res*. 2014;14:505. doi: 10.1186/s12913-014-0505-5 25344333 PMC4210514

[40] Axmon A, Björkman M, Ahlström G. Hospital readmissions among older people with intellectual disability in comparison with the general population. *J Intellect Disabil Res*. 2019;63(6):593–602. doi: 10.1111/jir.12601 30734976 PMC6850197

[41] Chang C-K, Chen C-Y, Broadbent M, Stewart R, O’Hara J. Hospital admissions for respiratory system diseases in adults with intellectual disabilities in Southeast London: a register-based cohort study. *BMJ open*. 2017;7(3):e014846. doi: 10.1136/bmjopen-2016-014846 28360254 PMC5372120

[42] Singhi P. Infectious causes of seizures and epilepsy in the developing world. *Dev Med Child Neurol*. 2011;53(7):600–9. doi: 10.1111/j.1469-8749.2011.03928.x 21518343

[43] Finlayson J, Morrison J, Jackson A, Mantry D, Cooper SA. Injuries, falls and accidents among adults with intellectual disabilities. Prospective cohort study. *J Intellect Disabil Res*. 2010;54(11):966–80. doi: 10.1111/j.1365-2788.2010.01319.x 21040056

[44] McDermott S, Royer J, Mann JR, Armour BS. Factors associated with ambulatory care sensitive emergency department visits for South Carolina Medicaid members with intellectual disability. *J Intellect Disabil Res*. 2018;62(3):165–78. doi: 10.1111/jir.12429 29027297 PMC5803329

