# Supplementary material for: Readmission and emergency department presentation after hospitalisation for epilepsy in people with intellectual disability: A data linkage study
Source: PLoS One. 2022 Aug 1;17(8):e0272439. doi: 10.1371/journal.pone.0272439 (PMC9342714; doi:10.1371/journal.pone.0272439)
Supplement: S1 Table — (DOCX) [file pone.0272439.s001.docx]

**Table S1. Variables and respective values used to define an unplanned hospitalisation/readmission.**

| Variable | Description | Value |
| --- | --- | --- |
| Emergency status | Indicates whether or not, in the opinion of the treating clinician, the admission was an emergency, that is, care or treatment was required within 24 hours. Applies to Public Hospital data only. | = Emergency |
| Episode of care type | This item is used to record the principal clinical intent or treatment goal of the care provided to the patient for the episode of care. | = Acute care |
