# Supplementary material for: Readmission and emergency department presentation after hospitalisation for epilepsy in people with intellectual disability: A data linkage study
Source: PLoS One. 2022 Aug 1;17(8):e0272439. doi: 10.1371/journal.pone.0272439 (PMC9342714; doi:10.1371/journal.pone.0272439)
Supplement: S2 Table — (DOCX) [file pone.0272439.s002.docx]

**Table S2. Baseline characteristics of children and adults, including those with incomplete data, with and without intellectual disability (n, %).**

|  | Children (N=2285) | |  | Adults (N=12476) | |  |
| --- | --- | --- | --- | --- | --- | --- |
|  | **ID**  **(N=781)** | **Non-ID (N=1504)** | **P value** | **ID**  **(N=2012)** | **Non-ID**  **(N=10464)** | **P value** |
| Male | 452 (57.9) | 803 (53.4) | 0.04 | 1143 (56.8) | 6023 (57.6) | 0.53 |
| Median (IQR) age (years) | 9.6  (7.1 – 12.4) | 10.8  (7.9 – 13.5) |  | 36.8  (24.9 – 48.4) | 39.7  (28.2 – 50.9) |  |
| Age range (years)2 |  |  |  |  |  |  |
| 16-24 | N/A | N/A |  | 509 (25.3) | 1929 (18.4) | <.0001 |
| 25-44 | N/A | N/A |  | 851 (42.3) | 4568 (43.6) |  |
| 45-64 | N/A | N/A |  | 652 (32.4) | 3967 (37.9) |  |
| Born in Australia | 737 (94.4) | 1373 (91.3) | 0.009 | 1870 (92.9) | 8371 (80.0) | <.0001 |
| Remoteness of residence |  |  |  |  |  |  |
| Major city | 522 (66.8) | 976 (64.9) | <.0001 | 1317 (65.5) | 6709 (64.1) | <.0001 |
| Inner regional | 171 (21.9) | 342 (22.7) |  | 476 (23.7) | 2321 (22.2) |  |
| Outer regional/remote/very remote | 83 (10.6) | 127 (8.4) |  | 205 (10.2) | 960 (9.2) |  |
| Unknown(missing) | 5 (0.6) | 59 (3.9) |  | 14 (0.7) | 474 (4.5) |  |
| Index of relative socio-economic disadvantage |  |  |  |  |  |  |
| 1 – 2 (most disadvantaged) | 157 (20.1) | 382 (25.5) | 0.0001 | 385 (19.1) | 2534 (24.2) | <.0001 |
| 3 – 4 | 157 (20.1) | 297 (19.8) |  | 411 (20.4) | 2191 (20.9) |  |
| 5 – 6 | 170 (21.8) | 288 (19.2) |  | 423 (21.0) | 1933 (18.5) |  |
| 7 – 8 | 157 (20.1) | 206 (13.7) |  | 438 (21.8) | 1654 (15.8) |  |
| 9 – 10 (least disadvantaged) | 115 (14.7) | 271 (18.0) |  | 297 (14.8) | 1671 (15.9) |  |
| Unknown(missing) | 25 (3.2) | 59 (3.9) |  | 58 (2.9) | 481 (4.6) |  |
| Characteristics of the index admission |  |  |  |  |  |  |
| Private health insurance |  |  |  |  |  |  |
| Yes | 183 (23.4) | 431 (28.7) | 0.004 | 213 (10.6) | 2109 (20.2) | <.0001 |
| No | 554 (76.6) | 1019 (71.3) |  | 1706 (84.8) | 7932 (75.8) |  |
| Unknown (missing) | 44 (5.6) | 54 (3.6) |  | 93 (4.6) | 423 (4.0) |  |
| Public hospital | 775 (99.2) | 1480 (98.4) | 0.10 | 1994 (99.1) | 10338 (98.8) | 0.23 |
| Median (IQR) length of stay (days) | 1.0  (1.0 – 2.0) | 1.0  (1.0 – 2.0) |  | 1.0  (1.0 – 3.0) | 1.0  (1.0 – 3.0) |  |
| Length of stay (days) |  |  |  |  |  |  |
| 1 – 2 | 601 (77.0) | 1285 (85.4) | <.0001 | 1388 (69.0) | 7779 (74.3) | <.0001 |
| 3 – 6 | 128 (16.4) | 184 (12.2) |  | 413 (20.5) | 1752 (16.7) |  |
| ≥7 | 52 (6.7) | 35 (2.3) |  | 211 (10.5) | 933 (8.9) |  |
| Mode of separation |  |  |  |  |  |  |
| Discharge by hospital | 780 (>99) | 1503 (>99) | >.99 | 1974 (98.1) | 10386 (99.3) | <.0001 |
| Transfer to nursing home | <5 (<1)^a^ | <5 (<1)^a^ |  | 19 (0.9) | 59 (0.6) |  |
| Transfer to other accommodation | 0 | 0 |  | 19 (0.9) | 19 (0.2) |  |
| ≥ one admission in the year prior | 403 (51.6) | 422 (28.1) | <.0001 | 959 (47.7) | 4428 (42.3) | <.0001 |
| Charlson Comorbidity Index |  |  |  |  |  |  |
| 0 | 737 (94.4) | 1447 (96.2) | 0.11 | 1751 (87.0) | 8782 (83.9) | <.0001 |
| 1 – 2 | 38 (4.9) | 51 (3.4) |  | 197 (9.8) | 1021 (9.8) |  |
| ≥3 | 6 (0.8) | 6 (0.4) |  | 64 (3.2) | 661 (6.3) |  |
| Psychiatric comorbidity^b^ | 292 (37.4) | 89 (5.9) | <.0001 | 576 (28.6) | 2905 (27.8) | 0.43 |

^a^ True value and/or percentage were censored to ensure confidentiality.

^b^ ICD-10 codes for psychiatric comorbidity: F00-F99, except for intellectual disability (F70-F79) and dementia (F00-F03 or F05.1; according to the codes included in the Charlson Comorbidity Index).
