# Supplementary material for: Readmission and emergency department presentation after hospitalisation for epilepsy in people with intellectual disability: A data linkage study
Source: PLoS One. 2022 Aug 1;17(8):e0272439. doi: 10.1371/journal.pone.0272439 (PMC9342714; doi:10.1371/journal.pone.0272439)
Supplement: Attachment — Submitted filename: Response to reviewers.docx [file pone.0272439.s003.docx]

Rebuttal letter

We would like to thank the academic editor and reviewers for their comprehensive and constructive comments; Table 1 documents each comment and our response to each comment.

**Table 1 Actions according to the editor and reviewers’ comments from PLOS Medicine**

| **No.** | **Comments** | **Response** |
| --- | --- | --- |
| **Academic editor** | | |
| 1.1 | how intellectual disability was defined and the severity of it is a key missing piece of information | Thank you for raising this issue up. We revised the ID definition in the introduction (the first sentence) and added citations, as per one reviewer’s comment:   - Page 4, line 52: “Intellectual disability, defined by impairments in cognitive and adaptive functioning with onset during childhood the developmental period” - Page 6, lines 105-109: “……by which people classified as having intellectual disability must have a diagnosis based on the Diagnostic and Statistical Manual of Mental Disorders IV or ICD-10.^1,2^ This strategy resulted in formation of a cohort of people with intellectual disability who needed medical or disability service supports in NSW, representing about 1% of the NSW population in 2015,^25^ comparable to the average estimate worldwide.^3^”   We did lack data to indicate the severity of ID, which is a limitation of the study. We have acknowledged this limitation in the discussion section:   - Page 19-20, lines 357-360: “While we were not able to ascertain disability severity, we have generated evidence on the needs of people with intellectual disability as a whole and identified areas that could benefit from further research.” |
| 1.2 | A significant amount of residual confounding:  The use of ICD-10 codes cannot capture the disability situation and the aggressiveness of treatment before or during the hospitalisation to control seizures | We agree that there are some residual confounding factors. However, intellectual disability situation is not a confounder, as the disability situation is only relevant to the cohort with intellectual disability. As mentioned in 1.1, we could not capture the disability information and have acknowledged the limitation and stated how the findings could benefit the community. This is also a common practice in comparative studies about the health service use of people with and without intellectual disability. ^1^ |
| 1.3 | Care for seizures and status epilepticus have advanced significantly from 2005 to 2016 and even 2020, and the outcomes should be analyzed by epochs | We agree that it might be better if we could analyse the data chronologically. However, it would be a different study design to answer different research questions. If dividing this current study population into groups according to the year of their index admission, we would not have enough cases for each year. |
| 1.4 | The age range of 16-64 is too broad, and there is likely also to be significant imbalances in medical conditions that could drive non-seizure readmissions | We confirm that we have controlled for age among the adult population in the regression model. To further clarify this, we have made some edits in the text to explain the choice of age limit and the relationship between age and service types:  Page 6, lines 98-102: “The age limits were chosen to avoid bias introduced by young children yet to receive an intellectual disability diagnosis and older people for whom our data capture may be incomplete due to transition to aged care services.^26^ Excluding older adults also reduced the likelihood of inclusion of epilepsy associated with late life disorders like dementia,^27^ which may complicate service use.”  Page 7, lines 127-129: “……outcomes, and the age of transition from paediatric to adult inpatient services in NSW, Australia.” |
| 1.5 | More details are needed about the non-seizure diagnosis to determine what could be the clinical interventions stemming from the study, such as pneumonia prevention, DVT treatment and prevention, more outpatient neurology and EMU referrals? | We have presented data for specific diagnoses for respiratory and neurological disorders. We have made some edits related to implications:  Page 18-19, lines 332-333: “……which should emphasise continuity of care in the community, more frequent reviews of management, and access to highly specialised reviews for those with complex epilepsy and health needs.” |
| 1.6 | Combining seizures and status epilepticus is too broad | We did not combine seizures and status epilepticus diagnosis to identify the index hospital admission. The index admission was defined by the diagnosis of epilepsy or status epilepticus. |
| 1.7 | It is not as clear the novelty in this manuscript, some of this work has been done elsewhere already | We have stated the novelty of this manuscript in the introduction and discussion:  Page 17, lines 292-293: “Expanding the previous knowledge, we further showed high readmission and ED presentation rates within 30……”  When available, we have compared our findings with the previous research (Page 17, lines 291-292). For the rest, to the best of our knowledge, there is no previous research answering the same research questions. |
| **Reviewer 1 – statistical reviewer** | | |
| 2.1 | Line numbers | Thanks for the advice. We have added line numbers. |
| 2.2 | If the authors considered a survival analysis, with time to readmission being the dependent variable. That might be more interesting or yield results that are more useful | Thanks for the suggestion. We did consider a survival analysis. However, there was a violation of the proportional hazard assumption. We have made some edits to clarify this:  Page 7, lines 137-138: “……a survival analysis could not be used because the proportional hazards assumption was violated.” |
| 2.3 | p. 3 Although I am a stats reviewer, I do know something about intellectual disability. I don’t think the definition in the introduction is adequate. You have to say something about severity. | Please refer to our response 1.1 |
| 2.4 | If possible, it would be nice to add whether there is an interaction between epilepsy and ID, or whether the increase in hospitalization is additive | Because everyone has epilepsy at the baseline, we could not do an interaction analysis between epilepsy and ID. |
| 2.5 | Excluding people with missing data may be problematic. How many people were there? What variables were they missing? In many situations, multiple imputation is a better solution than deletion | We did not do multiple imputations because, first, the proportion of individuals with missing data was small. Second, we have provided the characteristics of the participants with complete data, which suggested that the missing was at least close to being random (Page 9, lines 169-171; Table S2). We have made some edits to clarify the missing situation further:  Figure 1: added how many missing IDs and non-IDs, respectively  Page 8, lines 155-156: “In the main analysis, we excluded individuals with missing data (less than 10%), as the cohort characteristics before and after the exclusion were comparable. We also performed a sensitivity analysis to test the impact of excluding people with missing data.” |
| 2.6 | Please give more details on the matching procedure. Does the matching consist of just making strata? (That might be OK, but I wasn’t clear on whether this was what was done) | The matching procedure did not consist of making strata only. It also applied weight to each individual’s observations to account for the number of people in their strata. In our case, it was a coincidence that we did not need to exclude any individual after the matching procedure:  Page 6, lines 112-114: “We formed strata defined by age group (2-years for children <16 years and 5-years for adults) and sex, with a weight applied to each individual’s observations to account for the number of people in their strata.” |
| 2.7 | I commend the authors for not using any automated methods of variable selection and, instead, relying on their intelligence and expertise to do this | We would like to thank the review for this positive comment. |
| 2.8 | Table 1 should have a footnote saying what test was used to get the p value | Thank you for this suggestion. We have added the information (Table 1; Page 10-11) |
| 2.9 | Figure 2 - This might be better as a mosaic plot, depending on exactly what the authors are trying to show | Thank you for this suggestion. After a discussion, we decided to change to a segmented bar chart, which appeared more suitable for our data. The main difference between a mosaic plot and a segmented bar chart is that the latter can easily show the size of the population of each group. This feature was unnecessary in our case, as there were only two groups (people with and without intellectual disability) with different sample sizes. The segmented bar chart is a better choice to convey the key message (Figure 2). |
| **Reviewer 2** | | |
| 3.1 | It is unclear what kinds of hospitalization for epilepsy or readmission were excluded from the analyses. Same question goes to the term ‘unplanned’ admission. What kinds of planned admission were excluded? | Thank you for raising this question. We have added a supplementary file to present this information (Table S1). |
| 3.2 | In the introduction section, author stated that ‘However, this study relied solely on hospital records to identify intellectual disability status, probably resulting in a sample with more severe disability or complex health needs. 23 Further research is needed to help understand how intellectual disability affects readmission risk after epilepsy hospitalisation.’ It is unclear which study they were talking about when they referred to ‘this study replied solely on …’. If they referred to their own study, it is more suitable for the discussion section | We have revised the text to clarify this point:  Page 4, line 71: “However, this the aforementioned study relied solely on hospital records to identify intellectual disability status……” |
| 3.3 | The statement ‘The association between higher risk of acute care and intellectual disability was independent of sociodemographic, health, and index admission characteristics in this study. We surmise epilepsy 17 characteristics, and potentially inappropriate or inadequate management, may have driven the excess acute care in people with intellectual disability.’ Is it logic to say so? The former sentence stated that it was independent of index admission characteristics while the following sentence stated in opposite way | Thank you for raising this question. The factors mentioned in the latter part of the sentence (epilepsy characteristics and potentially inappropriate or inadequate management) refer to factors uncontrolled in our study. We have made some edits to the text to clarify this:  Page 18, lines 311-312: “We surmise that factors that have not been controlled for, including epilepsy characteristics, and potentially inappropriate or inadequate management……” |
| **Reviewer 3 – general comment:** I would support publication, with some clarification of the points around: population percentage with ID, ages for transition and whether there is analysis of the degree of ID as those with an F71-73 diagnosis have more healthcare utilisation than those with an F70 diagnosis. | | |
| 4.1 | Some assertions need more clarity, Without clarity these data are difficult to interpret   1. an example being that the prevalence of ID is given as 1-2% from meta-analysis, however, the use of the DSM and ICD criteria, would mean an IQ based prevalence, which is 3% (2 SD from the norm). 2. Another issue is that service provision in NSW is not described and an example would be transition to Adult services or to Later Life services. Some childrens services transition at 13, some at 16 and some at 18, depending on where in the world you practice.  Similarly, for later life services, some transition at 65, some at 75 - the assumption I have in this paper is that the transitions are 16 and 75, but this means that some children will then have been readmitted as adults - this isn’t clarified, whereas it is for the 65’s. | Thank you for the comments. For the first comment, the meta-analysis (Maulik et al., 2011) showed that the population pooled prevalence according to DSM/ICD criteria was 8.68/1000.^2^  We have made some edits for the second comment to clarify this; please refer to response 1.4. |
| 4.2 | It appears that the ID population in NSW is disadvantaged to start as the lower socioeconomic groups are over-represented.  The degree of ID also affects the health utilisation of services, often due to additional health needs due to their ID, there is no separation in the data between someone living in their own home with ID and someone requiring 24h care with their ID. | Thank you for raising these concerns. For socioeconomic status, our data showed that middle socioeconomic status was over-represented in people with intellectual disability than those without (Table 1). Additionally, we have controlled for socioeconomic status in the regression model.  For the comment related to ID severity, please refer to response 1.1.  We did not have information on the living arrangements. We have acknowledged the limitation in the discussion:  Page 20, lines 362-366: “Additionally, although the residential setting could influence ED visits in people with intellectual disability,^43^ we did not have complete data on the residential setting or living arrangements.” |
| 4.3 | By definition, those requiring more care will have a greater number of conditions and this may also require hospital admissions. The over-representation of respiratory admissions may have something to do with aspiration pneumonia, for example, more common in those with profound and multiple ID - this data doesn’t support this level of interrogation. | We acknowledged that our data did not support any interrogation by ID severity. However, we have investigated the details of respiratory admissions and provided some evidence of an overrepresentation of aspiration, flu, and pneumonia. |
| **Reviewer 4** | | |
| 5.1 | (On page 5) The first sentence of the introduction provides a definition of “intellectual disability” but the authors do not refer to a the major authoritative body such as the WHO or one of the major diagnostic manuals such as the DSM or ICD. Later, on page 5, the authors state that the diagnoses of intellectual disability were drawn from multiple health and disability datasets and do, in this instance, referred to the DSM and ICD. The authors should be consistent in their definition, which should be based on that provided by a major authority, as indicated here | Thank you for pointing out this issue. We have added the citations:  Page 4, line 52: citation 1-2. |
| 5.2 | It is noted that the information on the ED presentations covered only 60% of NSW public hospitals. The authors rightly mention this in the limitations section at the end. They should also consider how this limitation might have affected the results. | Thank you for this suggestion. With the incomplete coverage of EDs, we are likely to have under-estimated ED presentations. The effect of this underestimation should be minimal. We have made some edits to the text:  Page 20, lines 364-366: “Finally, while the participating EDs are large centres that serve a substantial proportion of the NSW population, the lack of population coverage of ED presentations means that we have underestimated ED presentations.^29^” |
| 5.3 | The authors have stated: “We also calculated the median day to the first outcome”. It is not clear what they mean by this. They should state their intended meaning more clearly. | We have made some edits to the text:  Page 7, line 134: “We also calculated the median number of days to the first outcome.” |
| 5.4 | Towards the end of the page the authors refer to “other neurodevelopmental conditions, which are rare in children without intellectual disability”. If attention deficit hyperactivity disorder and autism spectrum disorder are included in the definition of “neurodevelopmental conditions” these are not rare in children without intellectual disability. | Thank you for raising this concern.  The prevalence of neurodevelopmental conditions was lower in children without ID than in children with ID, which resulted in insufficient power for proper control. “Rare” is not the most appropriate word to use in this case. We have made some edits to the text:  Page 8, lines 153-154: “……less common in the general population and, more importantly, may lie on the causal pathway.”  As stated in the sentence, we did not include this variable in the model because neurodevelopment disorders were likely to lie on the causal pathway. |

The page and line numbers are based on the tracked version of the manuscript.

**Reference**

1.	Balogh R, Brownell M, Ouellette-Kuntz H, Colantonio A. Hospitalisation rates for ambulatory care sensitive conditions for persons with and without an intellectual disability--a population perspective. Journal of intellectual disability research : JIDR. 2010;54(9):820-32. doi: 10.1111/j.1365-2788.2010.01311.x.

2.	Maulik PK, Mascarenhas MN, Mathers CD, Dua T, Saxena S. Prevalence of intellectual disability: A meta-analysis of population-based studies. Res Dev Disabil. 2011;32(2):419-36. doi: <https://doi.org/10.1016/j.ridd.2010.12.018>.
